# 4-[(1-Benzyl-1,2,3-triazol-5-yl)meth­yl]-2*H*-1,4-benzo­thia­zin-3(4*H*)-one

**DOI:** 10.1107/S1600536814003900

**Published:** 2014-02-26

**Authors:** Nada Kheira Sebbar, Mohammed El Fal, El Mokhtar Essassi, Mohamed Saadi, Lahcen El Ammari

**Affiliations:** a Laboratoire de Chimie Organique Hétérocyclique URAC 21, Pharmacochimie, Avenue Ibn Battouta, BP 1014, Faculté des Sciences, Université Mohammed V-Agdal, Rabat, Morocco; b Laboratoire de Chimie du Solide Appliquée, Faculté des Sciences, Université Mohammed V-Agdal, Avenue Ibn Battouta, BP 1014, Rabat, Morocco

## Abstract

The asymmetric unit of the title compound, C_18_H_16_N_4_OS, contains two independent mol­ecules of similar conformation, the most relevant difference being the dihedral angle formed by the benzene rings [57.8 (2) and 52.7 (2)°]. The six-membered heterocycle of the benzo­thia­zine fragment exhibits a screw-boat conformation in both mol­ecules. The plane through the triazole ring is nearly perpendicular to those through the fused and terminal benzene rings [dihedral angles of 74.2 (2) and 83.2 (2)° in one mol­ecule, and 77.8 (2) and 82.9 (2)° in the other]. In the crystal, mol­ecules are linked by C—H⋯N and C—H⋯O hydrogen bonds into chains parallel to the *a-*axis direction. The crystal used was a non-merohedral twin, the refined ratio of twin components being 0.85 (10):15 (10).

## Related literature   

For the biological activity and pharmaceutical properties of benzo­thia­zines and their derivatives, see: Chia *et al.* (2008 ▶); Baraza­rte *et al.* (2008 ▶); Takemoto *et al.* (1994 ▶); Yaltirik *et al.* (2001 ▶). For a related structure, see: Sebbar *et al.* (2014 ▶). For ring puckering parameters, see: Cremer & Pople (1975 ▶).
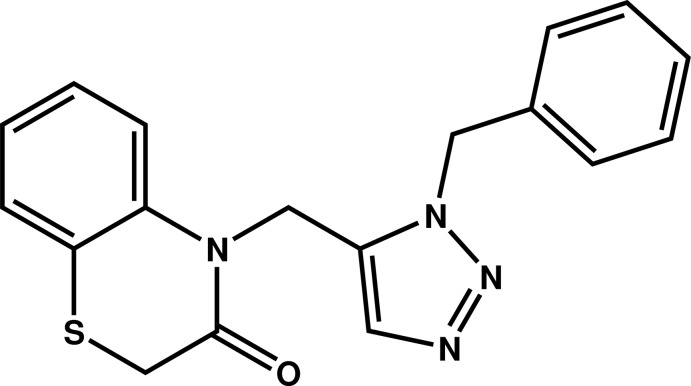


## Experimental   

### 

#### Crystal data  

C_18_H_16_N_4_OS
*M* *_r_* = 336.41
Orthorhombic, 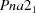

*a* = 34.5438 (15) Å
*b* = 6.0102 (2) Å
*c* = 15.6138 (7) Å
*V* = 3241.7 (2) Å^3^
*Z* = 8
Mo *K*α radiation
μ = 0.21 mm^−1^
*T* = 296 K
0.41 × 0.36 × 0.28 mm

#### Data collection  

Bruker X8 APEX diffractometer
Absorption correction: multi-scan (*SADABS*; Bruker, 2009 ▶) *T*_min_ = 0.645, *T*_max_ = 0.746
32914 measured reflections
6623 independent reflections
4634 reflections with *I* > 2σ(*I*)
*R*_int_ = 0.059

#### Refinement  

*R*[*F* ^2^ > 2σ(*F* ^2^)] = 0.062
*wR*(*F* ^2^) = 0.167
*S* = 1.04
6623 reflections
434 parameters
1 restraint
H-atom parameters constrained
Δρ_max_ = 0.61 e Å^−3^
Δρ_min_ = −0.23 e Å^−3^

### 

Data collection: *APEX2* (Bruker, 2009 ▶); cell refinement: *SAINT-Plus* (Bruker, 2009 ▶); data reduction: *SAINT-Plus*; program(s) used to solve structure: *SHELXS97* (Sheldrick, 2008 ▶); program(s) used to refine structure: *SHELXL97* (Sheldrick, 2008 ▶); molecular graphics: *ORTEP-3 for Windows* (Farrugia, 2012 ▶); software used to prepare material for publication: *PLATON* (Spek, 2009 ▶) and *publCIF* (Westrip,2010 ▶).

## Supplementary Material

Crystal structure: contains datablock(s) I. DOI: 10.1107/S1600536814003900/rz5106sup1.cif

Structure factors: contains datablock(s) I. DOI: 10.1107/S1600536814003900/rz5106Isup2.hkl

Supporting information file. DOI: 10.1107/S1600536814003900/rz5106Isup3.cml

CCDC reference: 987907

Additional supporting information:  crystallographic information; 3D view; checkCIF report

## Figures and Tables

**Table 1:** Hydrogen-bond geometry (Å, °)

*D*—H⋯*A*	*D*—H	H⋯*A*	*D*⋯*A*	*D*—H⋯*A*
C7—H7*A*⋯O2	0.97	2.56	3.452 (5)	153
C7—H7*B*⋯N3^i^	0.97	2.54	3.508 (5)	173
C17—H17*B*⋯N6^ii^	0.97	2.53	3.413 (5)	151
C25—H25*B*⋯N7^iii^	0.97	2.49	3.454 (5)	176

Symmetry codes: (i) 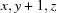; (ii) 
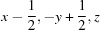
; (iii) 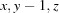.

**Fig. 1.**
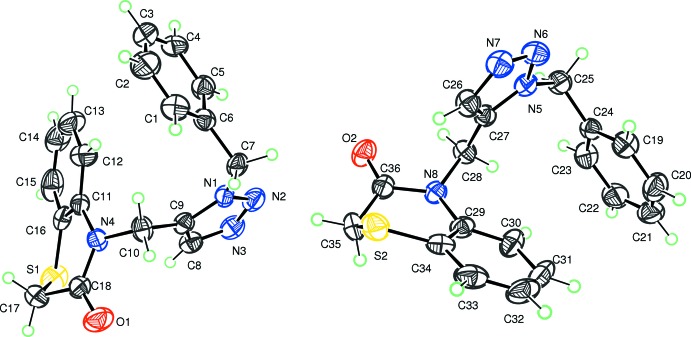
The molecular structure of the title compound with displacement ellipsoids drawn at the 50% probability level. H atoms are represented as small circles.

## supplementary crystallographic information

### 1. Comment

Benzothiazine derivatives have extensively been studied in different areas of chemistry including the pharmaceutical and other chemical industries. With respect to biological applications, these derivatives have been found to be potent anti-inflammatories (Chia *et al.*, 2008), anti-microbials (Barazarte *et al.*, 2008), herbicidals (Takemoto *et al.*, 1994) and fungicidals (Yaltirik *et al.*, 2001). The present work is a continuation of the investigation of the benzothiazine derivatives published recently by our team, including the isomer of the present compound (Sebbar *et al.*, 2014).

Each independent molecule of the title compound is build up from two fused six-membered rings linked to a triazol ring which is attached to a benzyl group as shown in Fig. 1. The 1,4-thiazine ring adopts a screw boat conformation as indicated by the puckering amplitude (S1/N4/C11/C16–C18) Q = 0.692 (4) Å, and spherical polar angle θ = 114.6 (3)°, with φ = 142.6 (4)°; (S2/N8/C29/C34–C36) Q = 0.676 (3) Å, and spherical polar angle θ = 64.3 (3)°, with φ = 321.0 (4)° (Cremer & Pople, 1975).

In one molecule (S1/O1/N1–N4/C1–C18), the triazol ring (N1/N2/N3/C8/C9) makes dihedral angles of 74.2 (2)° and 83.2 (2)°, with the benzene fused to the 1,4-thiazine ring (C11–C16) and the other benzene ring (C1–C6), respectively. Moreover, the dihedral angle between the two benzene rings is of 57.8 (2)°. Nearly the same values are observed in the second molecule (S2/O2/N5–N8/C19–C36; 77.8 (2)°; 82.9 (2)° and 52.7 (2)°). The most important differences between the conformations of the present molecule and its isomer, recently published, are the values of the dihedral angles between the benzene rings. In the crystal, the molecules are linked by intermolecular C–H···N and C–H···O hydrogen interactions (Table 1) to form wavy chains parallel to the *a* axis.

### 2. Experimental

A mixture of 4-(prop-2-yn-1-yl)-3,4-dihydro-2*H*-1,4-benzothiazin-3-one (0.27 g, 0.35 mmol) and benzylazide (1.34 ml, 0.35 mmol) in ethanol (5 ml) was stirred at room temperature for 24 h. After cooling, the solid obtained was purified by column chromatography on silica gel with ethyl acetate-hexane (1:2 *v*/*v*) as eluent. Crystals were isolated when the solvent was allowed to evaporate at room temperature.

### 3. Refinement

All H atoms were located in a difference Fourier map and treated as riding, with C—H = 0.93–0.97 Å and with *U*_iso_(H) = 1.2 *U*_eq_(C). The crystal used for the diffraction study was a non-merohedral twin (twin law 1 0 0, 0 -1 0, 0 0 -1) with a 0.85 (10): 15 (10) domain ratio. The reported Flack parameter was obtain by the TWIN/BASF procedure in SHELXL-97 (Sheldrick, 2008)

### Crystal data

**Table:**

C_18_H_16_N_4_OS	*F*(000) = 1408
*M**_r_* = 336.41	*D*_x_ = 1.379 Mg m^−^^3^
Orthorhombic, *P**n**a*2_1_	Mo *K*α radiation, λ = 0.71073 Å
Hall symbol: P 2c -2n	Cell parameters from 6623 reflections
*a* = 34.5438 (15) Å	θ = 1.2–26.4°
*b* = 6.0102 (2) Å	µ = 0.21 mm^−^^1^
*c* = 15.6138 (7) Å	*T* = 296 K
*V* = 3241.7 (2) Å^3^	Block, colourless
*Z* = 8	0.41 × 0.36 × 0.28 mm

### Data collection

**Table:**

Bruker X8 APEX diffractometer	6623 independent reflections
Radiation source: fine-focus sealed tube	4634 reflections with *I* > 2σ(*I*)
Graphite monochromator	*R*_int_ = 0.059
φ and ω scans	θ_max_ = 26.4°, θ_min_ = 1.2°
Absorption correction: multi-scan (*SADABS*; Bruker, 2009)	*h* = −43→43
*T*_min_ = 0.645, *T*_max_ = 0.746	*k* = −5→7
32914 measured reflections	*l* = −19→18

### Refinement

**Table:**

Refinement on *F*^2^	Primary atom site location: structure-invariant direct methods
Least-squares matrix: full	Secondary atom site location: difference Fourier map
*R*[*F*^2^ > 2σ(*F*^2^)] = 0.062	Hydrogen site location: inferred from neighbouring sites
*wR*(*F*^2^) = 0.167	H-atom parameters constrained
*S* = 1.04	*w* = 1/[σ^2^(*F*_o_^2^) + (0.0979*P*)^2^] where *P* = (*F*_o_^2^ + 2*F*_c_^2^)/3
6623 reflections	(Δ/σ)_max_ < 0.001
434 parameters	Δρ_max_ = 0.61 e Å^−^^3^
1 restraint	Δρ_min_ = −0.23 e Å^−^^3^

### Special details

**Table:**

Geometry. All e.s.d.'s (except the e.s.d. in the dihedral angle between two l.s. planes) are estimated using the full covariance matrix. The cell e.s.d.'s are taken into account individually in the estimation of e.s.d.'s in distances, angles and torsion angles; correlations between e.s.d.'s in cell parameters are only used when they are defined by crystal symmetry. An approximate (isotropic) treatment of cell e.s.d.'s is used for estimating e.s.d.'s involving l.s. planes.
Refinement. Refinement of *F*^2^ against all reflections. The weighted *R*-factor *wR* and goodness of fit *S* are based on *F*^2^, conventional *R*-factors *R* are based on *F*, with *F* set to zero for negative *F*^2^. The threshold expression of *F*^2^ > σ(*F*^2^) is used only for calculating *R*-factors(gt) *etc*. and is not relevant to the choice of reflections for refinement. *R*-factors based on *F*^2^ are statistically about twice as large as those based on *F*, and *R*- factors based on all data will be even larger.

### Fractional atomic coordinates and isotropic or equivalent isotropic displacement parameters (Å^2^)

**Table:**

	*x*	*y*	*z*	*U*_iso_*/*U*_eq_	
C1	0.31692 (12)	0.4325 (6)	0.1447 (3)	0.0545 (11)	
H1	0.3094	0.5530	0.1782	0.065*	
C2	0.31671 (15)	0.4529 (8)	0.0576 (4)	0.0704 (14)	
H2	0.3091	0.5860	0.0323	0.084*	
C3	0.32759 (13)	0.2787 (9)	0.0081 (4)	0.0687 (14)	
H3	0.3270	0.2924	−0.0512	0.082*	
C4	0.33941 (13)	0.0825 (8)	0.0443 (3)	0.0628 (12)	
H4	0.3473	−0.0357	0.0100	0.075*	
C5	0.33950 (11)	0.0624 (6)	0.1323 (3)	0.0509 (10)	
H5	0.3474	−0.0707	0.1572	0.061*	
C6	0.32804 (11)	0.2370 (6)	0.1843 (3)	0.0417 (10)	
C7	0.32913 (12)	0.2159 (6)	0.2803 (3)	0.0462 (10)	
H7A	0.3556	0.1892	0.2986	0.055*	
H7B	0.3206	0.3545	0.3059	0.055*	
C8	0.25772 (13)	−0.1859 (6)	0.3428 (3)	0.0478 (10)	
H8	0.2332	−0.2464	0.3508	0.057*	
C9	0.26532 (10)	0.0219 (5)	0.3095 (3)	0.0394 (8)	
C10	0.23912 (10)	0.2045 (6)	0.2791 (3)	0.0459 (10)	
H10A	0.2341	0.3077	0.3255	0.055*	
H10B	0.2515	0.2856	0.2328	0.055*	
C11	0.20359 (10)	−0.0363 (6)	0.1767 (2)	0.0398 (8)	
C12	0.22918 (13)	0.0036 (7)	0.1102 (3)	0.0605 (11)	
H12	0.2441	0.1323	0.1100	0.073*	
C13	0.23244 (16)	−0.1462 (10)	0.0451 (4)	0.0809 (16)	
H13	0.2487	−0.1146	−0.0008	0.097*	
C14	0.21204 (18)	−0.3443 (10)	0.0458 (4)	0.0811 (17)	
H14	0.2162	−0.4519	0.0041	0.097*	
C15	0.18533 (15)	−0.3777 (8)	0.1101 (4)	0.0730 (15)	
H15	0.1701	−0.5053	0.1091	0.088*	
C16	0.18064 (11)	−0.2289 (6)	0.1749 (3)	0.0453 (10)	
C17	0.13479 (12)	0.0198 (7)	0.2700 (3)	0.0652 (12)	
H17A	0.1263	0.0833	0.2160	0.078*	
H17B	0.1142	0.0377	0.3115	0.078*	
C18	0.17070 (11)	0.1349 (6)	0.3005 (3)	0.0469 (9)	
C19	0.58380 (12)	0.4007 (6)	0.6217 (3)	0.0507 (10)	
H19	0.5916	0.5348	0.5976	0.061*	
C20	0.58469 (14)	0.3719 (8)	0.7105 (4)	0.0634 (13)	
H20	0.5925	0.4893	0.7453	0.076*	
C21	0.57425 (12)	0.1738 (8)	0.7474 (3)	0.0630 (13)	
H21	0.5760	0.1538	0.8063	0.076*	
C22	0.56089 (13)	0.0029 (7)	0.6947 (3)	0.0621 (12)	
H22	0.5528	−0.1306	0.7191	0.075*	
C23	0.55945 (11)	0.0290 (6)	0.6070 (3)	0.0513 (10)	
H23	0.5506	−0.0866	0.5725	0.062*	
C24	0.57126 (10)	0.2294 (5)	0.5702 (3)	0.0374 (9)	
C25	0.57129 (12)	0.2524 (6)	0.4742 (3)	0.0476 (11)	
H25A	0.5976	0.2764	0.4547	0.057*	
H25B	0.5620	0.1150	0.4489	0.057*	
C26	0.50118 (12)	0.6660 (6)	0.4162 (3)	0.0477 (10)	
H26	0.4770	0.7326	0.4105	0.057*	
C27	0.50797 (10)	0.4601 (5)	0.4493 (2)	0.0354 (8)	
C28	0.48073 (10)	0.2863 (5)	0.4814 (3)	0.0413 (9)	
H28A	0.4752	0.1801	0.4363	0.050*	
H28B	0.4924	0.2069	0.5290	0.050*	
C29	0.44632 (11)	0.5389 (5)	0.5809 (2)	0.0399 (9)	
C30	0.47044 (13)	0.4886 (6)	0.6500 (3)	0.0524 (10)	
H30	0.4837	0.3541	0.6519	0.063*	
C31	0.47433 (16)	0.6435 (9)	0.7163 (3)	0.0686 (14)	
H31	0.4900	0.6086	0.7628	0.082*	
C32	0.45615 (16)	0.8412 (9)	0.7149 (3)	0.0707 (15)	
H32	0.4603	0.9443	0.7583	0.085*	
C33	0.43120 (13)	0.8897 (7)	0.6482 (3)	0.0592 (12)	
H33	0.4178	1.0239	0.6484	0.071*	
C34	0.42577 (11)	0.7403 (6)	0.5807 (3)	0.0446 (10)	
C35	0.37906 (11)	0.5172 (6)	0.4797 (3)	0.0542 (11)	
H35A	0.3683	0.4549	0.5318	0.065*	
H35B	0.3594	0.5113	0.4354	0.065*	
C36	0.41402 (11)	0.3868 (5)	0.4525 (3)	0.0409 (9)	
N1	0.30457 (8)	0.0352 (4)	0.3104 (2)	0.0390 (7)	
N2	0.31989 (10)	−0.1534 (5)	0.3427 (2)	0.0535 (9)	
N3	0.29094 (11)	−0.2861 (5)	0.3615 (3)	0.0555 (10)	
N4	0.20276 (8)	0.1095 (4)	0.2491 (2)	0.0401 (7)	
N5	0.54672 (9)	0.4385 (5)	0.4449 (2)	0.0399 (7)	
N6	0.56333 (10)	0.6217 (5)	0.4108 (2)	0.0506 (8)	
N7	0.53574 (10)	0.7587 (5)	0.3928 (3)	0.0506 (9)	
N8	0.44458 (8)	0.3937 (4)	0.50956 (19)	0.0367 (7)	
O1	0.17165 (10)	0.2455 (5)	0.3667 (3)	0.0691 (10)	
O2	0.41597 (9)	0.2844 (4)	0.3865 (2)	0.0570 (8)	
S1	0.14594 (4)	−0.2667 (2)	0.25665 (9)	0.0732 (4)	
S2	0.39377 (3)	0.79990 (17)	0.49747 (8)	0.0568 (3)	

### Atomic displacement parameters (Å^2^)

**Table:**

	*U*^11^	*U*^22^	*U*^33^	*U*^12^	*U*^13^	*U*^23^
C1	0.060 (3)	0.045 (2)	0.059 (3)	−0.0026 (17)	0.005 (2)	0.0008 (19)
C2	0.074 (3)	0.070 (3)	0.067 (4)	0.001 (2)	0.004 (3)	0.021 (3)
C3	0.056 (3)	0.102 (4)	0.048 (3)	−0.001 (3)	−0.001 (2)	0.016 (3)
C4	0.055 (3)	0.089 (3)	0.044 (3)	0.007 (2)	0.010 (2)	−0.016 (2)
C5	0.049 (2)	0.051 (2)	0.052 (3)	0.0045 (17)	−0.001 (2)	−0.0030 (19)
C6	0.0362 (19)	0.0440 (18)	0.045 (3)	−0.0080 (15)	0.0001 (18)	−0.0056 (16)
C7	0.046 (2)	0.056 (2)	0.036 (3)	−0.0117 (17)	−0.0081 (18)	−0.0084 (17)
C8	0.058 (3)	0.0445 (19)	0.041 (2)	0.0044 (17)	0.002 (2)	−0.0004 (17)
C9	0.045 (2)	0.0394 (17)	0.034 (2)	−0.0005 (14)	−0.0036 (17)	−0.0054 (15)
C10	0.042 (2)	0.0408 (18)	0.055 (3)	−0.0035 (15)	−0.0070 (19)	−0.0036 (17)
C11	0.037 (2)	0.0474 (18)	0.035 (2)	0.0070 (15)	−0.0046 (17)	−0.0024 (16)
C12	0.060 (3)	0.071 (3)	0.051 (3)	0.000 (2)	0.008 (2)	−0.003 (2)
C13	0.084 (4)	0.113 (4)	0.045 (3)	0.029 (3)	0.004 (3)	−0.012 (3)
C14	0.099 (4)	0.090 (4)	0.055 (4)	0.026 (3)	−0.018 (3)	−0.032 (3)
C15	0.079 (3)	0.060 (3)	0.081 (4)	0.006 (2)	−0.034 (3)	−0.023 (3)
C16	0.049 (2)	0.0470 (19)	0.040 (3)	0.0022 (16)	−0.016 (2)	−0.0030 (16)
C17	0.041 (2)	0.102 (3)	0.053 (3)	−0.001 (2)	0.004 (2)	−0.001 (3)
C18	0.048 (2)	0.054 (2)	0.039 (2)	0.0079 (17)	−0.0021 (19)	−0.0021 (18)
C19	0.054 (2)	0.0416 (18)	0.057 (3)	−0.0028 (16)	0.000 (2)	−0.0030 (18)
C20	0.064 (3)	0.069 (3)	0.057 (3)	0.004 (2)	−0.012 (2)	−0.018 (2)
C21	0.056 (3)	0.088 (3)	0.044 (3)	0.017 (2)	0.005 (2)	0.013 (3)
C22	0.057 (3)	0.071 (3)	0.058 (3)	−0.004 (2)	0.003 (2)	0.026 (2)
C23	0.049 (2)	0.0446 (19)	0.060 (3)	−0.0015 (16)	−0.005 (2)	0.0013 (18)
C24	0.0345 (19)	0.0371 (16)	0.041 (3)	0.0065 (14)	0.0008 (17)	0.0044 (15)
C25	0.045 (2)	0.0491 (19)	0.049 (3)	0.0112 (16)	0.0013 (19)	−0.0002 (17)
C26	0.046 (2)	0.0428 (18)	0.054 (3)	0.0065 (16)	−0.004 (2)	0.0080 (18)
C27	0.037 (2)	0.0363 (15)	0.033 (2)	0.0030 (13)	−0.0005 (15)	−0.0024 (14)
C28	0.040 (2)	0.0343 (15)	0.050 (3)	0.0043 (14)	0.0010 (18)	0.0017 (16)
C29	0.045 (2)	0.0394 (17)	0.035 (2)	−0.0078 (15)	0.0049 (17)	0.0024 (15)
C30	0.063 (3)	0.052 (2)	0.042 (3)	−0.0080 (18)	−0.011 (2)	0.0059 (19)
C31	0.086 (4)	0.086 (3)	0.034 (3)	−0.033 (3)	−0.012 (2)	0.002 (2)
C32	0.087 (4)	0.079 (3)	0.046 (3)	−0.035 (3)	0.015 (3)	−0.028 (3)
C33	0.064 (3)	0.053 (2)	0.061 (3)	−0.0151 (19)	0.023 (3)	−0.018 (2)
C34	0.049 (2)	0.0431 (17)	0.042 (3)	−0.0002 (16)	0.0155 (19)	−0.0027 (16)
C35	0.036 (2)	0.068 (2)	0.059 (3)	0.0004 (18)	−0.009 (2)	−0.001 (2)
C36	0.043 (2)	0.0403 (16)	0.040 (2)	−0.0063 (15)	−0.0034 (18)	0.0043 (17)
N1	0.0372 (17)	0.0434 (14)	0.0364 (18)	0.0033 (12)	−0.0053 (14)	−0.0031 (13)
N2	0.063 (2)	0.0571 (18)	0.041 (2)	0.0148 (17)	−0.0057 (17)	0.0015 (16)
N3	0.070 (2)	0.0505 (18)	0.046 (3)	0.0011 (17)	−0.0018 (19)	0.0076 (16)
N4	0.0350 (16)	0.0415 (14)	0.0437 (19)	0.0027 (12)	−0.0031 (14)	−0.0066 (14)
N5	0.0453 (19)	0.0398 (14)	0.0346 (18)	0.0024 (12)	−0.0012 (14)	0.0017 (13)
N6	0.052 (2)	0.0502 (17)	0.050 (2)	−0.0095 (15)	0.0064 (17)	0.0097 (16)
N7	0.059 (2)	0.0418 (15)	0.051 (3)	−0.0060 (15)	−0.0006 (18)	0.0095 (14)
N8	0.0411 (16)	0.0348 (13)	0.0342 (17)	0.0004 (11)	−0.0022 (14)	−0.0016 (13)
O1	0.085 (2)	0.0756 (19)	0.047 (2)	0.0087 (17)	0.0052 (18)	−0.0166 (15)
O2	0.066 (2)	0.0606 (15)	0.044 (2)	−0.0043 (14)	−0.0107 (16)	−0.0147 (14)
S1	0.0715 (8)	0.0856 (8)	0.0625 (9)	−0.0403 (6)	−0.0057 (7)	0.0059 (7)
S2	0.0524 (6)	0.0563 (5)	0.0617 (8)	0.0170 (4)	0.0037 (6)	0.0024 (5)

### Geometric parameters (Å, º)

**Table:**

C1—C2	1.366 (7)	C19—H19	0.9300
C1—C6	1.382 (6)	C20—C21	1.370 (7)
C1—H1	0.9300	C20—H20	0.9300
C2—C3	1.354 (7)	C21—C22	1.394 (7)
C2—H2	0.9300	C21—H21	0.9300
C3—C4	1.370 (7)	C22—C23	1.380 (7)
C3—H3	0.9300	C22—H22	0.9300
C4—C5	1.380 (7)	C23—C24	1.396 (5)
C4—H4	0.9300	C23—H23	0.9300
C5—C6	1.384 (5)	C24—C25	1.504 (7)
C5—H5	0.9300	C25—N5	1.477 (5)
C6—C7	1.506 (7)	C25—H25A	0.9700
C7—N1	1.456 (5)	C25—H25B	0.9700
C7—H7A	0.9700	C26—C27	1.361 (5)
C7—H7B	0.9700	C26—N7	1.368 (5)
C8—N3	1.329 (5)	C26—H26	0.9300
C8—C9	1.378 (5)	C27—N5	1.347 (4)
C8—H8	0.9300	C27—C28	1.493 (5)
C9—N1	1.358 (4)	C28—N8	1.473 (4)
C9—C10	1.500 (5)	C28—H28A	0.9700
C10—N4	1.457 (4)	C28—H28B	0.9700
C10—H10A	0.9700	C29—C30	1.396 (5)
C10—H10B	0.9700	C29—C34	1.403 (5)
C11—C12	1.384 (6)	C29—N8	1.416 (4)
C11—C16	1.403 (5)	C30—C31	1.399 (7)
C11—N4	1.431 (5)	C30—H30	0.9300
C12—C13	1.363 (7)	C31—C32	1.344 (7)
C12—H12	0.9300	C31—H31	0.9300
C13—C14	1.384 (8)	C32—C33	1.382 (7)
C13—H13	0.9300	C32—H32	0.9300
C14—C15	1.378 (9)	C33—C34	1.398 (6)
C14—H14	0.9300	C33—H33	0.9300
C15—C16	1.360 (7)	C34—S2	1.743 (5)
C15—H15	0.9300	C35—C36	1.501 (5)
C16—S1	1.766 (5)	C35—S2	1.795 (4)
C17—C18	1.498 (6)	C35—H35A	0.9700
C17—S1	1.777 (5)	C35—H35B	0.9700
C17—H17A	0.9700	C36—O2	1.202 (5)
C17—H17B	0.9700	C36—N8	1.382 (5)
C18—O1	1.230 (5)	N1—N2	1.349 (4)
C18—N4	1.376 (5)	N2—N3	1.312 (5)
C19—C24	1.377 (6)	N5—N6	1.351 (4)
C19—C20	1.398 (8)	N6—N7	1.290 (4)
			
C2—C1—C6	121.5 (5)	C23—C22—C21	120.9 (4)
C2—C1—H1	119.2	C23—C22—H22	119.6
C6—C1—H1	119.2	C21—C22—H22	119.6
C3—C2—C1	119.8 (5)	C22—C23—C24	119.7 (4)
C3—C2—H2	120.1	C22—C23—H23	120.1
C1—C2—H2	120.1	C24—C23—H23	120.1
C2—C3—C4	120.9 (6)	C19—C24—C23	119.8 (4)
C2—C3—H3	119.6	C19—C24—C25	120.9 (4)
C4—C3—H3	119.6	C23—C24—C25	119.3 (4)
C3—C4—C5	119.1 (5)	N5—C25—C24	112.2 (3)
C3—C4—H4	120.4	N5—C25—H25A	109.2
C5—C4—H4	120.4	C24—C25—H25A	109.2
C4—C5—C6	121.1 (4)	N5—C25—H25B	109.2
C4—C5—H5	119.4	C24—C25—H25B	109.2
C6—C5—H5	119.4	H25A—C25—H25B	107.9
C1—C6—C5	117.5 (5)	C27—C26—N7	108.8 (3)
C1—C6—C7	121.6 (4)	C27—C26—H26	125.6
C5—C6—C7	120.8 (4)	N7—C26—H26	125.6
N1—C7—C6	111.7 (3)	N5—C27—C26	103.8 (3)
N1—C7—H7A	109.3	N5—C27—C28	125.2 (3)
C6—C7—H7A	109.3	C26—C27—C28	130.9 (3)
N1—C7—H7B	109.3	N8—C28—C27	109.1 (3)
C6—C7—H7B	109.3	N8—C28—H28A	109.9
H7A—C7—H7B	107.9	C27—C28—H28A	109.9
N3—C8—C9	109.2 (4)	N8—C28—H28B	109.9
N3—C8—H8	125.4	C27—C28—H28B	109.9
C9—C8—H8	125.4	H28A—C28—H28B	108.3
N1—C9—C8	103.9 (3)	C30—C29—C34	119.4 (3)
N1—C9—C10	124.2 (3)	C30—C29—N8	120.0 (3)
C8—C9—C10	131.9 (4)	C34—C29—N8	120.5 (3)
N4—C10—C9	109.6 (3)	C29—C30—C31	119.0 (4)
N4—C10—H10A	109.8	C29—C30—H30	120.5
C9—C10—H10A	109.8	C31—C30—H30	120.5
N4—C10—H10B	109.8	C32—C31—C30	122.1 (5)
C9—C10—H10B	109.8	C32—C31—H31	118.9
H10A—C10—H10B	108.2	C30—C31—H31	118.9
C12—C11—C16	119.2 (4)	C31—C32—C33	119.3 (4)
C12—C11—N4	120.0 (3)	C31—C32—H32	120.3
C16—C11—N4	120.7 (3)	C33—C32—H32	120.3
C13—C12—C11	119.9 (5)	C32—C33—C34	121.1 (4)
C13—C12—H12	120.1	C32—C33—H33	119.5
C11—C12—H12	120.1	C34—C33—H33	119.5
C12—C13—C14	121.3 (5)	C33—C34—C29	119.0 (4)
C12—C13—H13	119.3	C33—C34—S2	121.0 (3)
C14—C13—H13	119.3	C29—C34—S2	120.0 (3)
C15—C14—C13	118.2 (5)	C36—C35—S2	108.1 (3)
C15—C14—H14	120.9	C36—C35—H35A	110.1
C13—C14—H14	120.9	S2—C35—H35A	110.1
C16—C15—C14	121.7 (5)	C36—C35—H35B	110.1
C16—C15—H15	119.2	S2—C35—H35B	110.1
C14—C15—H15	119.2	H35A—C35—H35B	108.4
C15—C16—C11	119.4 (4)	O2—C36—N8	121.7 (3)
C15—C16—S1	122.3 (4)	O2—C36—C35	123.7 (4)
C11—C16—S1	118.3 (3)	N8—C36—C35	114.6 (3)
C18—C17—S1	107.8 (3)	N2—N1—C9	110.2 (3)
C18—C17—H17A	110.2	N2—N1—C7	121.3 (3)
S1—C17—H17A	110.2	C9—N1—C7	128.5 (3)
C18—C17—H17B	110.2	N3—N2—N1	107.2 (3)
S1—C17—H17B	110.2	N2—N3—C8	109.5 (3)
H17A—C17—H17B	108.5	C18—N4—C11	123.0 (3)
O1—C18—N4	121.9 (4)	C18—N4—C10	117.6 (3)
O1—C18—C17	122.6 (4)	C11—N4—C10	118.4 (3)
N4—C18—C17	115.5 (4)	C27—N5—N6	111.3 (3)
C24—C19—C20	119.6 (4)	C27—N5—C25	128.9 (3)
C24—C19—H19	120.2	N6—N5—C25	119.7 (3)
C20—C19—H19	120.2	N7—N6—N5	107.0 (3)
C21—C20—C19	121.2 (4)	N6—N7—C26	109.1 (3)
C21—C20—H20	119.4	C36—N8—C29	123.9 (3)
C19—C20—H20	119.4	C36—N8—C28	116.2 (3)
C20—C21—C22	118.7 (5)	C29—N8—C28	117.9 (3)
C20—C21—H21	120.6	C16—S1—C17	96.1 (2)
C22—C21—H21	120.6	C34—S2—C35	95.77 (19)

### Hydrogen-bond geometry (Å, º)

**Table:**

*D*—H···*A*	*D*—H	H···*A*	*D*···*A*	*D*—H···*A*
C7—H7*A*···O2	0.97	2.56	3.452 (5)	153
C7—H7*B*···N3^i^	0.97	2.54	3.508 (5)	173
C17—H17*B*···N6^ii^	0.97	2.53	3.413 (5)	151
C25—H25*B*···N7^iii^	0.97	2.49	3.454 (5)	176

Symmetry codes: (i) *x*, *y*+1, *z*; (ii) *x*−1/2, −*y*+1/2, *z*; (iii) *x*, *y*−1, *z*.
